# Bloom Filter Approach for Autonomous Data Acquisition in the Edge-Based MCS Scenario †

**DOI:** 10.3390/s22030879

**Published:** 2022-01-24

**Authors:** Martina Antonić, Aleksandar Antonić, Ivana Podnar Žarko

**Affiliations:** 1 Faculty of Electrical Engineering and Computing, University of Zagreb, Unska 3, 10000 Zagreb, Croatia; ivana.podnar@fer.hr; 2 Croatian Lottery, Ul. Grada Vukovara 72, 10000 Zagreb, Croatia; aleksandar.antonic@lutrija.hr

**Keywords:** mobile crowdsensing, Bloom filter, multi-access edge computing, mobile edge computing

## Abstract

Mobile crowdsensing (MCS) is a sensing paradigm that allows ordinary citizens to use mobile and wearable technologies and become active observers of their surroundings. MCS services generate a massive amount of data due to the vast number of devices engaging in MCS tasks, and the intrinsic mobility of users can quickly make information obsolete, requiring efficient data processing. Our previous work shows that the Bloom filter (BF) is a promising technique to reduce the quantity of redundant data in a hierarchical edge-based MCS ecosystem, allowing users engaging in MCS tasks to make autonomous informed decisions on whether or not to transmit data. This paper extends the proposed BF algorithm to accept multiple data readings of the same type at an exact location if the MCS task requires such functionality. In addition, we thoroughly evaluate the overall behavior of our approach by taking into account the overhead generated in communication between edge servers and end-user devices on a real-world dataset. Our results indicate that using the proposed algorithm makes it possible to significantly reduce the amount of transmitted data and achieve energy savings up to 62% compared to a baseline approach.

## 1. Introduction

The Internet of Things (IoT) reached the peak of expectations, according to Gartner in 2016 [1], and we are witnessing a consolidation of the developed technologies and paradigms beyond initial trials and prototype solutions. IoT platforms are increasingly deployed to support and connect many heterogeneous IoT devices and store and continuously process the generated data streams. Thus, the digitalization of our everyday environment resulted in a vast number of novel IoT services addressing the needs of citizens (e.g., monitoring of personal pollution exposure or live traffic data). Eventually, this has evolved into a concept called mobile crowdsensing (MCS), which utilizes many users to create knowledge from data generated by moving devices with various sensing capabilities without requiring the deployment of a particular physical infrastructure. Guo et al. proposed a formal definition of MCS as *“a new sensing paradigm that empowers ordinary citizens to contribute data sensed or generated from their mobile devices, aggregates, and fuses the data in the cloud for crowd intelligence extraction and people-centric service delivery”* [2]. In other words, users carry mobile sensing devices while moving through the city to become a rich source of contextual information about their environment. Although citizens sense local environments, the collective usage of sensed data can enable dense spatiotemporal sensing coverage and create awareness about specific large-scale phenomena, thus improving life quality and knowledge about their local community [3]. A wide range of MCS applications that rely on the power of the crowd to collectively sense the environment and share data of common interest have been used across several domains, from environmental and traffic monitoring to healthcare and social networks [4]. The main goal of such applications is to extract knowledge about the sensed phenomena by using different data analytic techniques and informing citizens about their surroundings, which can affect their decisions and behavior while being mobile. Due to the large number of devices that can be active in parallel, the MCS ecosystem must efficiently process data. At the same time, it also has to quickly disseminate information to interested users, as their context changes frequently, and information can become stale if it is not delivered as soon as it becomes available. Furthermore, users’ inherent mobility leads to dynamic sensing coverage of geographical areas, where some areas may be densely covered with measurements. In contrast, others may remain unexplored or with insufficient data readings, posing additional challenges for data acquisition and device management [5].

Due to the volatile volumes of sensor data, in the beginning, a cloud infrastructure seemed suitable for the implementation of an elastic, scalable, and modular MCS ecosystem. All components responsible for processing integrated data streams were located in the cloud, while various data sources and consumers were deployed on mobile devices with connections to the Internet, as presented in [6]. Even though cloud-based architecture provides enormous computing capabilities and storage capacity, continuous processing of incoming data streams might become relatively inefficient in large-scale MCS ecosystems when there are numerous concurrent connections between user devices and cloud servers. Data processing and business intelligence are eventually brought closer to end-users to resolve these issues. With the help of 5G technology, which adds powerful processing capabilities to the radio access network [7], the MCS ecosystem design nowadays shifts to a distributed architecture where the cloud service is only responsible for coordinating processing components deployed at the network edge. Multi-access edge computing (MEC), previously known as mobile edge computing, has already been acknowledged as a critical enabler of improved context-aware services that move computationally demanding tasks previously executed on the central server in mobile users’ proximity, thus lowering data propagation latency and bandwidth consumption. In our previous work [8], we proposed an edge-based architecture for MCS. In such distributed environment, mobile edge (ME) servers, placed between physical sensing devices and the cloud, take responsibility for device management in their geographical area. In particular, they keep track of available (and connected) devices, monitor sensor data acquisition, and aggregate received data readings before sending a final result to the cloud. Such hierarchical processing reduces the amount of raw data that is transmitted over the network, as edge MCS components (i.e., ME hosts) control the data acquisition process in their deployment area by providing instructions to mobile devices on whether to send their data readings or not.

In this paper, we address the problem of data optimization, which entails reducing the amount of data transmitted over the network while also providing adequate data coverage following application requirements because collecting and transmitting all data is resource-intensive and usually not necessary. Specifically, we present a distributed algorithm based on the Bloom filter (BF) data structure, which tries to filter redundant data close to its origin and thus reduce the amount of transmitted raw data as much as feasible. By using the BF structure, we enable mobile devices to autonomously decide whether to collect and transmit data readings or not, without the need for cloud-based supervision and coordination. More specifically, each end-user device can independently decide whether sensed data is redundant (and should be discarded) or valuable (and should be sent to the ME host for further processing) based on its BF data structure filled with sent data readings. At the same time, the edge MCS service performs data aggregation, keeps the BF structure up-to-date, and disseminates BF to mobile devices located in its deployment area when it recognizes that a data source is not synced. The BF data structure carries information about data readings obtained from physically collocated devices (i.e., located in the same geographical area), and it does not provide a significant overhead in terms of processing, memory, and bandwidth consumption. An initial version of our BF-based distributed algorithm was introduced in [9]. In contrast to the previous publication, this paper includes the following new contributions:

- We present an extended version of the BF algorithm which can accept multiple data readings of the same type at the same location, both at the end-user device and ME host, as otherwise, the data production will be immediately stopped on all devices due to a BF structure inherent characteristics (i.e., inserted elements cannot be removed from a BF structure), to allow to compare similar data readings from multiple sources if MCS task requires such functionality in the edge-based distributed MCS environment.
- We outline the main procedures of the proposed BF algorithm.
- We run the same experiments as in [9] to evaluate BF regarding filter size and probability of false positives and analyze the number of lost data readings on a new and significantly larger real-world dataset collected in Seoul, South Korea [10], and confirm our initial findings.
- We include novel evaluation results on the same dataset to understand the overall behavior of the proposed BF approach by considering the overhead generated in communication between edge host and end-user devices when a user changes its location or sends redundant data reading.

Our results on a real-world dataset indicate that a BF is a valuable data structure to orchestrate autonomous data acquisition on users participating in MCS tasks. In particular, we show that using the proposed BF algorithm makes it possible to obviate redundant user activity and achieve energy savings up to 62% compared to a baseline approach requiring all generated data. The main advantage of the proposed hierarchical architecture is that there is no central point of failure as the data processing is distributed across multiple ME hosts, and all end-user devices can autonomously decide when to transmit data. At the same time, the actual energy savings depends on a specific MCS application and its setup.

The remainder of this article is organized as follows. First, in Section 2, we briefly survey related work addressing hierarchical fog and MEC solutions focusing on energy-efficient sensing, and the usage of Bloom filter across different domains. Then, we explain in detail a distributed edge-based architecture for MCS and present an algorithm that enables mobile devices to control the data acquisition process autonomously in Section 3. Finally, we extensively evaluate the applicability of the proposed approach and overhead introduced by filter control messages on a real-world dataset in Section 4 and conclude the paper in Section 5.

## 2. Related Work

This section gives a brief overview of the issues discussed in the paper. We first present notable works from the literature related to the hierarchical edge-based MCS environment, then energy-efficient sensing techniques in MCS, and, finally, the applicability of BF data structure across different applications domains.

### 2.1. Edge-Based MCS Environments

In recent years, edge computing has been recognized as a promising approach to facilitate data processing and reduce the amount of raw data transmitted to the cloud servers in distributed MCS environments. One of the early works presented in [11] is Mobile Edge Capture and Analytics (MECA), a middleware for data collection from mobile devices, in which an edge layer is used to receive requirements from the back-end servers, manage the data collection among a subset of local devices, and run edge analytics for primitive data processing. RedEdge is a novel big data processing solution that enables the processing of big data streams near the data source in mobile edge cloud computing environments [12]. In contrast, ref. [13] focuses on preserving privacy and dealing with malicious participants in edge computing enhanced MCS systems. Bonomi et al. proposed Fog Computing [14], a hierarchical and distributed platform that provides computational resources and storage to enable new services at the network edge. At the same time, Jayaraman et al. presented a distributed data analytics platform for MCS applications in which a fog layer is responsible for local computing and data storage [15]. Luan et al. [16] presented a three-tier Mobile–Fog–Cloud architecture that deploys highly virtualized computing and communication facilities at the proximity of mobile users. In contrast, Tang et al. [17] proposed a four-layer hierarchical distributed fog computing architecture that enables parallel data processing at the edge of the network and is, therefore, suitable for deploying smart city services. Another relevant approach described in [18] proposed an IoT-enabled MCS framework based on the oneM2M standard architecture. In contrast, in [19], Bellavista et al. proposed human-driven edge computing (HEC) as a new model to ease the provisioning and extend traditional MEC coverage solutions. They further extended their work in [20] by introducing the concept of social MEC (SMEC) proxies in the MCS environment, i.e., they add SMEC nodes between other devices and cloud, based on the incentives and centrality measures concerning other people in the group. In [21], the authors focused on the synergy between MCS and MEC and considered a possible extension of the HEC architecture by introducing mobile edge nodes operated by the users’ devices that can be selected as substitutes for fixed edge servers. In particular, they proposed a probabilistic model to estimate the number of nodes that need to be promoted as mobile MEC nodes to assist the MCS data gathering. Chen et al. [22] proposed a three-layer MCS architecture in which edge servers are used to process raw data, protect users’ privacy and improve response time, and propose a pricing incentive mechanism to address the problem that users may not actively participate in completing tasks by maximizing social welfare. Similarly, in [23], the authors proposed ParticipAct, an edge-enabled MCS platform that uses edge nodes to identify possible emergency crowd scenarios and deal with users’ rewards using a federated blockchain network. In contrast to the identified papers, which mainly deal with data processing at the network edge, in this paper, we focus on sensing process management to reduce the amount of redundant data transmitted over the network without the need for additional user actions.

### 2.2. Energy-Efficient Sensing in a Distributed MCS Environment

Liu et al. [24] proposed to leverage emerging deep reinforcement learning techniques for directing multiple mobile devices to collect data in a target area for MCS while ensuring geographical fairness and minimizing the energy consumption in a fully distributed manner. In [25], the authors proposed a new computing paradigm, named Edge Mesh, which distributes the decision-making tasks among edge devices within the network instead of sending all data to a centralized server, while in [26], the authors used game theory principles to select tasks and manage their scheduling on the network edge. Valerio et al. [27] considered a fog-based architecture for MCS and analyzed the trade-off between accuracy and consumed energy for collecting data from IoT sensors and performing distributed data analytics on mobile nodes passing by IoT devices, in addition to fog gateways at the network edge. They have shown that it is possible to significantly reduce the system’s energy consumption by using some short-range communication technologies while maintaining a satisfactory accuracy compared to a centralized cloud solution. Similarly, Alenazi et al. [28] aimed to minimize the total power consumption of the network by using a distributed machine learning approach in which the processing can take place in intermediary devices such as IoT nodes and fog servers in addition to the cloud. Another interesting approach is presented in [29], where authors proposed a distributed algorithm for adaptive scheduling of the video sensor node’s activity that enables each node to decide when to enter a sleep mode based on the neighbors’ activity, without compromising the coverage of its monitored region. A work presented in [30] introduced a distributed data collection framework for opportunistic MCS systems intending to minimize the sensing cost and data delivery to the participants while at the same time maximizing data collection utility. Similar to ours, the proposed mechanism prevents users from contributing too much data, and each device can decide the timing and duration of the sensing process.

### 2.3. Bloom Filter

In this paper, we focus on the applicability of BF data structure in the MCS domain, i.e., to determine whether a data item is already submitted to the system or not. The BF structure is well-known and widely used among different domains, especially for efficient testing of whether an item exists in a set. For example, Bloom filters are extensively used in spell-checking software to determine if the word is a member of the particular language [31], or they can be used for bar code recognition and processing [32]. Furthermore, they are applied for distributed search and caching in the web [33] and P2P environments [34], where the main goal is to find the exact location of resources, while we aim to determine whether the data is being produced on some of the devices at a specific location and time. In addition, one interesting approach is presented in [35], where BF is used to ensure efficient and secure matching of sensing users and tasks on end-user and fog nodes, while we want to ensure that end-user devices can autonomously decide to transmit only valuable data through the network. Xue et al. [36] tried to achieve secure and fine-grained MCS data sharing in which forward secrecy is based on the BF data structure. Similarly, Jinbo et al. [37] dealt with the MCS data privacy and proposed a BF-based user-union matching scheme to protect it, while Siddiqui et al. [38] proposed a BF-based secure data provenance mechanism suitable for resource-constrained devices in IoT networks. A secure cloud storage service for an IoT environment is presented in [39], where BF is used to verify the integrity of data saved in the cloud. The context-aware addressing and routing method described in [40] uses BFs along the routing protocol to express context information inside the IoT domain. In [41], the authors proposed a fog-based new technique for distributing certificate revocation information across IoT devices in which BF is used to reduce the size of the revocation list while maintaining an acceptable overhead. Another approach closely related to ours is presented in [42], where the authors employed BF to minimize the amount of data transmitted during the tag identification in large-scale RFID-based IoT systems. Amoretti et al. [43] proposed a distributed naming service for the IoT that relies on BFs to generate compact names from node descriptions, while Singh et al. [44] proposed the Accommodative Bloom filter approach to deal with massive data streaming from IoT sensor devices. In [45], the authors deal with an efficient broadcasting mechanism in IoT networks. In particular, they used BFs to prevent IoT nodes from being flooded with unwanted packets previously received. The concept most similar to ours is presented in [46], where BFs are used to identify the items for data dissemination in wireless sensor networks without global topology information. Additionally, BFs are used for packet routing and forwarding to improve network router performance [47], to increase network security (e.g., virus detection [48]), and in many other applications.

## 3. Materials and Methods

This section describes an edge-based architecture for MCS and algorithms used to achieve autonomous data acquisition in such a distributed environment. More precisely, we first present an edge-based decentralized MCS ecosystem. Then, we give a brief overview of the Bloom filter data structure that we use to communicate existing sensor readings between data sources and edge MCS services. Then, we present an algorithm that runs on mobile devices and enables them to autonomously control the data acquisition process by monitoring their local filter structure and deciding whether a reading is valuable to the system or not. Finally, we outline the main algorithm procedures.

### 3.1. Decentralized Edge-Based MCS Environment

MCS takes advantage of the widespread availability of mobile devices to monitor a variety of phenomena of common interest in which citizens can act as both data sources and consumers. An MCS ecosystem consists of two categories of users who communicate through an MCS service: those who are interested in a specific data (*requesters*) and those who can create the data of interest (*workers*), where the interest in sensor data can be seen as an MCS *task*. We denote workers as data sources in the MCS ecosystem. Data sources can be both humans and devices, typically mobile, and change geographical location over time. The data produced by workers needs to be geo-tagged, either by a precise location determined by GPS or cell identifier (e.g., a mobile network cell or MGRS area identifier—Military Grid Reference System (MGRS) is the geo-coordinate standard used by the North Atlantic Treaty Organisation (NATO) for locating points on the Earth.) as location represents the essential contextual information of the worker’s result (e.g., sensor data reading). At the same time, mobile devices can be used by task requesters, denoted as data consumers, to define new tasks and to receive the data of interest from the cloud. In general, entities in the MCS ecosystem interact as follows: A requester creates a new task, which is forwarded to the MCS service responsible for locating at least one eligible worker capable of collecting data for this task. Each task has related conditions (the geographical region of interest, required sensors, incentives, etc.) that must be met within a particular time frame, or the task will expire. The MCS service disseminates the task to all potential workers who can accept it and begin the sensing process if a good match exists between workers’ available sensors and task requirements. After collecting data, workers transmit their results to the MCS service, which aggregates all incoming data and disseminates it to interested parties (i.e., task requesters) in near real-time. As mobile devices usually provide limited filtering and aggregation mechanisms, workers generally deliver all raw data readings to the MCS service, governing the data production process. In addition, worker mobility patterns often result in a dynamic sensing coverage, with some locations extensively covered by data readings and others suffering from a lack of available data. Furthermore, the demand for MCS data fluctuates throughout time, so certain places may not require data coverage at all. Therefore, in order to conduct the data acquisition process in an energy- and bandwidth-efficient manner while preserving MCS application quality requirements (in terms of required data accuracy and frequency of data readings), intelligent data acquisition methods need to be applied in the MCS ecosystem to optimize the number of collected data readings.

**Figure 1:**
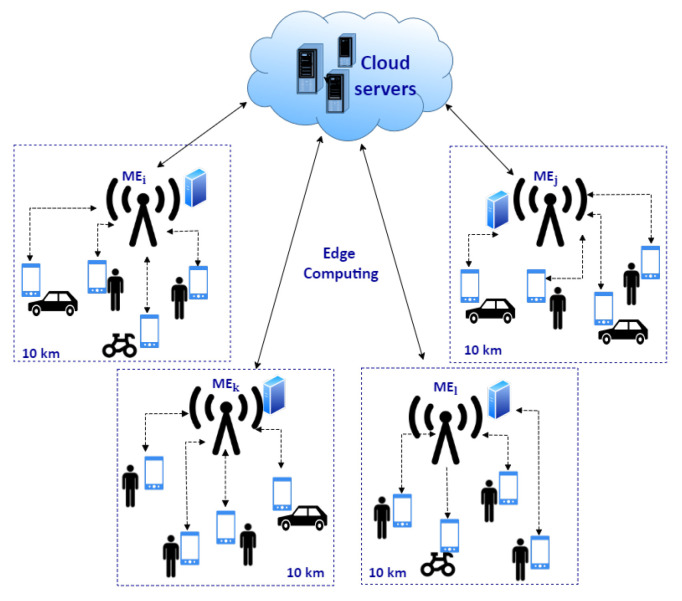
Decentralized edge-enabled MCS architecture.

In our previous [8] work, we have shown that MCS services can be decentralized by using multi-access edge computing (MEC) principles and moving computation in the proximity of mobile users. In such architecture, edge servers, located between physical sensing devices and the cloud, take responsibility for the management and coordination of MCS users in the geographical area under their supervision, as shown in Figure 1. Such distributed hierarchical edge-based MCS ecosystem consists of one cloud coordinator, several ME hosts deployed in different regions of interest, and multiple end-users acting as both workers and requesters in the system. The cloud coordinator has global knowledge about the MCS ecosystem because it knows all users and ME hosts connected to the system and all existing tasks. When a new task is submitted to the system, the coordinator forwards it to the corresponding ME host(s) based on its geographical area of interest, so an ME host knows all active tasks in its deployment area. Similarly, the first time a new worker enters the system, the coordinator looks for an ME host in the geographical region where the worker is currently situated. If there were no users in this area before, the coordinator dynamically deploys a new ME host in the area. Each ME host is responsible for one square-shaped geographical area with a side length of 10 km which has a unique MGRS identifier. Afterward, the worker announces his/her presence and capabilities (i.e., type of sensed data) to the corresponding host, so an MCS service operating on this ME host is aware of all possible workers in its deployment area and their capabilities. As a result, the ME host will assign active tasks to the new worker only if he/she can potentially complete them, thus preventing the worker’s device overload. Then, the new worker can start the sensing process and submit data to the MCS service running on the corresponding ME host. ME hosts have substantial computational power compared to mobile devices, so they can perform data aggregation and processing of all data produced by workers under their responsibility, disseminate results and control the data acquisition by managing all workers in the area. Each ME host sends the aggregated data for its deployment area to the cloud coordinator, who performs different analytics and correlates received data to extract knowledge about the sensed phenomena for a larger area.

### 3.2. Bloom Filter

To enable autonomous decision-making of a mobile device regarding its participation in sensor data acquisition, processing, and transmission to both edge and cloud servers, we have used a well-known Bloom filter (BF) approach. BF is a probabilistic data structure invented by Burton Howard Bloom in 1970 and is used to determine whether an element belongs to a set or not [49]. The fundamental feature of this structure is that it has a low rate of false positives, claiming that an element is part of the set when it is not, but never false negatives, stating that an element was not in the set when it was inserted [50]. A BF is an array of *m* bits that represents a set $S=\{r_{1},r_{2},\cdots ,r_{n}\}$ of *n* elements, which are all initially set to 0. The idea is to evenly map elements of a set to random integers in the range 1,⋯m by using *k* independent hash functions, $h_{i}\left(r\right),1\leq i\leq k$. In the context of MCS, an element is each data reading produced by the end-user device, and the set represents readings created by all devices in an *km* MGRS area. To encode data reading in the BF, we use the information about a measured property and location where this reading was produced. We need to discover the bits $h_{i}\left(r\right)$ that should be set to one for 1≤i≤k to add a reading (i.e., an element) *r* in a filter (and submit it to the ME host, so that the host can process it and disseminate it to all interested requesters). To test whether reading has previously been submitted to the ME host, we need to see if it has been encoded in a BF. To phrase it another way, we need to compute the *k* hash functions over the element and check if all bits in the array’s relevant places are set to 1. If at least one bit is set to 0, the reading *r* is valuable as it almost certainly does not exist in the ME host. If all bits are 1, either *r* is already sent to the ME host, or bits were set to 1 during the insertion of other elements, resulting in a false positive. The number of hash functions determines the likelihood of a false positive *p*, the number of added elements, and the length of the BF, where *k* is typically a constant much smaller than *m* and proportional to *n*. To calculate *p*, we first assume that each array position is chosen with equal probability by a hash function. As a result, if a BF has *m* bits and *k* hash functions, the probability that a bit is not set to 1 by any of the hash functions is (see Equation (1)):

(1) $$p_{\left(b=0\right)}={\left(1-\frac{1}{m}\right)}^{k},$$

where $p_{\left(b=0\right)}$ is the probability that a bit is 0, *m* is the number of bits, and *k* is the number of independent hash functions in a BF structure. After inserting *n* elements to the filter, the probability that a certain bit is still 0 is (see Equation (2)):

(2) $$p_{b=0}^{\prime }={\left(1-\frac{1}{m}\right)}^{kn},$$

where $p_{\left(b=0\right)}^{\prime }$ is the probability that a bit is 0 after the insertion of *n* elements, while the probability that it is 1 is (see Equation (3)):

(3) $$p_{b=1}^{\prime }=1-{\left(1-\frac{1}{m}\right)}^{kn},$$

where $p_{\left(b=1\right)}^{\prime }$ is the probability that a bit is 1 after the insertion of *n* elements. To decide if a reading has already been produced (i.e., an element belongs to a set), we must determine whether or not all of the *k* array positions in the filter computed by the hash functions are set to 1. When the element is not part of the set, the probability of this happening is (see Equation (4)):

(4) $$p={\left(1-{\left(1-\frac{1}{m}\right)}^{kn}\right)}^{k}\approx {\left(1-e^{-kn/m}\right)}^{k},$$

where *p* is the probability of false positives, i.e., all of the *k* array positions in BF are set to 1. That is, as the number of bits *m* increases, the chance of false positives drops and grows when the number of inserted items *n* increases. We have to minimize Equation (4) to get the optimal number of hash functions *k*. As a result, the value of *k* that minimizes the false positive probability *p* for a given *m* and *n* is (see Equation (5)):

(5) $$k=\frac{m}{n}\ln2.$$

The number of bits in a BF *m* must expand linearly with the number of elements inserted in the filter *n* to maintain a stable likelihood of false positives. As a result, for the desired number of items *n* and false positive probability *p*, the filter length *m* is (see Equation (6)):

(6) $$m=-\frac{n\ln p}{{\left(\ln2\right)}^{2}}.$$

We can calculate the number of hash functions *k* and filter size *m* based on those parameters using Equations (4)–(6), as the expected number of different readings that must be inserted into a BF can be obtained from MCS service requirements and a priori known data, and we can set the desired probability of false positives *p* for an MCS service.

Removing an element from a BF is not possible because false negatives are not permitted. To remove an element from a filter, we need to set any of $h_{i}\left(r\right)$ bits to 0. However, if other elements in a filter are mapped on that bit, they would also be removed, introducing the possibility for false negatives. This BF characteristic is in line with the MCS behavior, as only readings that have already been sent to an ME host are encoded in the Bloom filter (and processed by the ME host), and therefore they cannot be revoked.

### 3.3. Decentralized BF Algorithm

**Figure 2:**
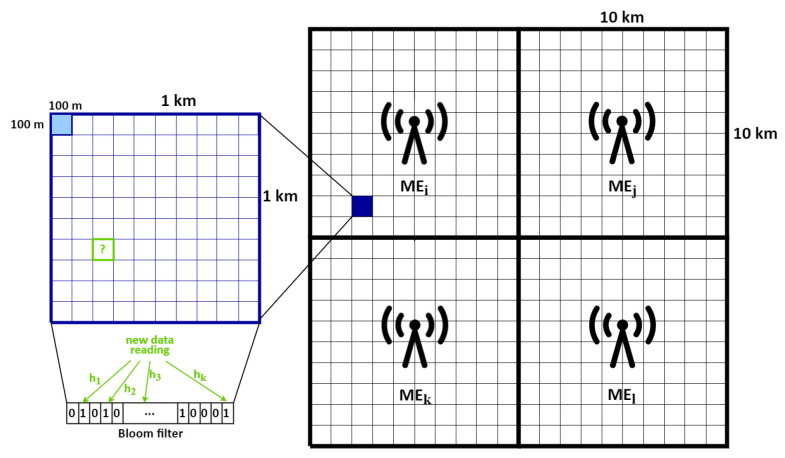
A setup for decentralized energy-efficient MCS from an ME host’s perspective.

The algorithm’s fundamental idea is to utilize a BF membership test on a mobile node to determine whether or not a new data reading should be reported to the ME host in the period under consideration. As already stated in Section 3.1 in the proposed hierarchical edge-based MCS ecosystem, each ME host is responsible for one square-shaped MGRS area with a side length of 10 km. As this is a pretty large deployment area, it is unlikely that a single user (i.e., worker) can cover it in a short period. Therefore, a worker’s geolocation is mapped to an MGRS area of 1 square kilometer (referred to as a *km* MGRS area from now on) where a user is currently located, depicted as the dark blue cell in Figure 2. In cases when redundant workers are available in cells of interest, we need only a subset of workers to collect and transmit data, while others may be deactivated to achieve battery savings and reduce unnecessary data reporting. Each worker maintains a BF structure for the *km* MGRS area where it is currently located, while an ME host takes care of all BFs in its corresponding MGRS area with a side length of 10 km. When a user enters in a new *km* area and *announces* his/her capabilities to the corresponding ME host, in the *assign* message, he/she will receive the active tasks and current state of the BF for this area. Note that each *km* MGRS area is associated with the one BF structure, which is created and maintained by the ME host and represents the union of BFs of all workers in that *km* MGRS area. The proposed architecture uses a BF structure both on mobile nodes and edge servers to minimize redundant worker activity while at the same time keeping uniform data density within the area.

In more detail, each worker queries its local filter structure to choose whether or not to produce more results in the area where it is now positioned. If the filter already contains data readings of the same type (note that it does not have to be the same value, but the same measured property) in the same MGRS area with a side length of 100 m in the observed period (shown as the light blue cell in Figure 2), data production will be suspended. Note that from now on, we call this 100 × 100 m^2^ cell the *small* MGRS area. (We opted for a *small* MGRS area in the process of encoding produced data readings because we want to collect data with greater density and equally distributed within a *km* MGRS area.) The data reading should be discarded if a BF membership test results positively. Otherwise, it is valuable, and a worker should transmit this reading to the ME host. In addition, a worker inserts the combination of reading data type and the ID of the *small* MGRS area where data was produced in its BF. Since such procedure would immediately stop a data acquisition on a worker node for this *small* area after submitting the first valuable result for a required data type, a worker can be instructed to produce multiple results of the same type in this area before updating its BF structure. That can be achieved by using a special variable $k_{s}$, indicating the required number of results per *small* area. Analogously, when the corresponding ME host receives a new data reading, it will use its BF to determine whether the reading is redundant (shown as “new data reading” which falls in the green cell in Figure 2). If the filter membership test is negative, the ME host will add the combination of reading data type and ID of the *small* MGRS area where the reading was created to its filter, ensuring a uniform density of data readings inside a *km* MGRS area. This procedure enables the synchronization between the two filters (i.e., the ME host’s and worker’s BFs). If the filter membership test reports positive, then the worker’s BF structure is inconsistent, and the ME host will send the current state of its BF to the worker that produced this data reading in a *BFupdate* message. In other words, the worker’s filter is out-of-date and has to be updated, as the submitted reading is redundant in this period. An ME host can be configured to require multiple results of the same type in the *small* MGRS area before updating its BF structure, but after a certain amount of time, the data production will be stopped on all workers as inserted elements cannot be removed from a BF structure. Therefore, a BF maintained by an ME host must be periodically reset and delivered to possible workers in the area in a *BFreset* message to avoid a scenario in which a filter membership test always finds that a received reading is redundant. Note that by sending the reset filter structure to favored workers first, an ME host can preference some workers based on predetermined parameters. The BF’s validity period (i.e., the time between two consecutive filter resets) is strongly dependent on the type of MCS service and can be defined in the configuration prior to the deployment.

**Figure 3:**
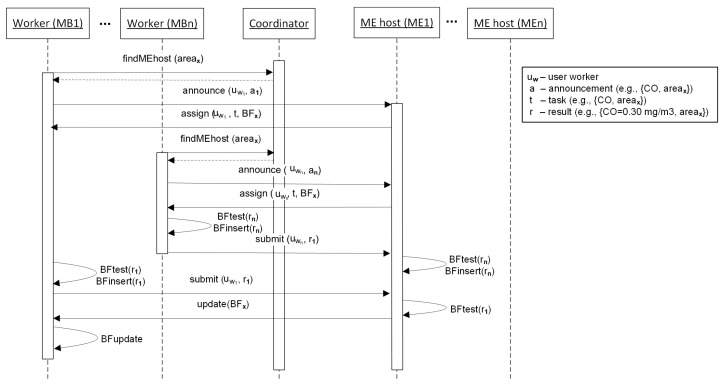
An algorithm for autonomous decision-making of a moving worker regarding its participation in the data acquisition process.

Figure 3 illustrates a scenario in which two workers enter the system and ask the coordinator about the ME host responsible for their geographical location. As they are in the same *km* MGRS area, the coordinator directs them to the $ME_{1}$ host. After announcing their capabilities to the $ME_{1}$ host, it finds currently available tasks to which they can potentially answer and assigns those tasks to workers, together with the current state of the BF for the area. First, worker $u_{w_{n}}$ produces a result and queries its BF structure to decide whether to transmit this result to the $ME_{1}$ host or not. As this is the first result in the area, the filter membership test reports negative, and $u_{w_{n}}$ inserts $r_{n}$ in the filter and submits $r_{n}$ to the $ME_{1}$ host. After receiving the result, the $ME_{1}$ host queries its filter, decides that this result is not redundant, and inserts $r_{n}$ in the filter. At the same time, worker $u_{w_{1}}$ produces a new result $r_{1}$ and queries its BF structure to decide whether to transmit this result to the edge server or not. As its filter is not up-to-date, it decides that $r_{1}$ should be submitted to the $ME_{1}$ host, although it is redundant. Upon receiving $r_{1}$, the host realizes that the worker’s filter should be refreshed and sends the current filter state to $u_{w_{1}}$. Afterwards, worker $u_{w_{1}}$ updates its BF.

### 3.4. Main Algorithm Procedures

Hereafter, we briefly explain five main procedures in our algorithm. First, in Algorithm 1, we show a procedure when a worker $u_{w}$ arrives in a *km* MGRS area operated by the $ME_{i}$ host. $ME_{i}$ identifies an area in which $u_{w}$ is currently located (line 2) and checks if he/she can potentially answer to any task in that area (lines 3–7). If any potential tasks are available, $ME_{i}$ sends a BF for this area and assigns all potential tasks to the worker $u_{w}$ (lines 8–13).

Next, we present a procedure when a worker $u_{w}$ submits a new result *r* to the $ME_{i}$ host in Algorithm 2. Before sending the data, a worker $u_{w}$ has to check if a similar result has already been produced in a *small* area of 100 m^2^ by querying the Bloom filter associated with the *km* MGRS area where he/she is currently located received from the corresponding ME host. Note that from now on, we call this worker’s BF. If a filter membership test reports positive, the result *r* contains redundant data and should be skipped (lines 4–6). Otherwise, the produced result should be sent to the $ME_{i}$ host. Worker $u_{w}$ submits data until a sufficient number of results are produced for this *small* MGRS area and adds this result in his/her BF (lines 7–13).
**Algorithm 1** Arrival of a new worker in the area covered by the ME host1:**procedure**Worker Announce($u_{w},a$)2:    $area\leftarrow findArea(\ell_{a})$3:    $workersInArea\left(area\right).add\left(u_{w}\right)$4:    **for each** τ in activeTasks(area) **do**5:        **if** $\mu_{\tau }\left(a\right)==⊤$ **then**6:           $potentialTasks\left(u_{w}\right).add\left(\tau \right)$7:        **end if**8:    **end for**9:    **if** $potentialTasks(u_{w})==⌀$ **then**10:        **break**11:    **else**12:        sendBF(bloomFilter(area),m)13:        $assign(u_{w},potentialTasks\left(u_{w}\right))$14:    **end if**15:**end procedure**

**Algorithm 2** Submission of a new result to the ME host by a worker *w*
1:**procedure**Submit Result($u_{w},r$)2:    $smallArea\leftarrow findArea(\ell_{r})$3:    property←getProperty(r)4:    **if** workerBloomFilter.membershipTest(smallArea+property) **then**5:        **break**6:    **else**7:        **if** $resultsCounter\left(smallArea+property\right)<k_{s}$ **then**8:           resultsCounter(smallArea+property)++9:           submitResult(r)10:        **else**11:           submitResult(r)12:           workerBloomFilter.add(smallArea+property)13:        **end if**14:    **end if**15:
**end procedure**



Algorithm 3 shows a procedure when the $ME_{i}$ host receives a new result *r* submitted by the worker $u_{w}$. First, $ME_{i}$ identifies an area in which $u_{w}$ is currently located (line 2) and analyzes features of a produced result (lines 3–4). Then, it checks whether its BF for the corresponding *km* MGRS area contains an element with the same combination of data type and *small* MGRS area as *r*. If yes, the result *r* contains redundant data, and the $ME_{i}$ host has to update the BF of the worker $u_{w}$ (lines 5–7). Otherwise, the submitted result is new and should be stored by the $ME_{i}$ host and delivered to the interested users. When the $ME_{i}$ host collects sufficient number of results *k* per *small* MGRS area, it adds this result in its BF (lines 8–13). In Algorithm 2, a worker maintains the resultsCounter, while in Algorithm 3, the resultsCounter is maintained by an ME host. An ME host will first reach the counter threshold (*k*) as it collects all measurements in the *small* MGRS area (from multiple workers), and then it will send the filled BF structure to all workers who reported redundant data reading, because the worker’s counter still did not reach the threshold value ($k_{s}$). Note that when an ME host accepts multiple data readings, a detection or filtering mechanism can be built on top of an ME host because it receives numerous readings per *small* MGRS area, and it can perform preprocessing of received data readings (e.g., to detect faulty values).
**Algorithm 3** Arrival of a new result in the area covered by the ME host1:**procedure**Receive Result($u_{w},r$)2:    $area\leftarrow findArea(\ell_{r})$3:    $smallArea\leftarrow findArea(\ell_{r})$4:    property←getProperty(r)5:    **if** bloomFilter(area).membershipTest(smallArea+property) **then**6:        $sendBF(bloomFilter\left(area\right),k_{s})$7:    **else**8:        **if** resultsCounter(smallArea+property)<k **then**9:           resultsCounter(smallArea+property)++10:           results(smallArea+property).add(r)11:        **else**12:           bloomFilter(area).add(smallArea+property)13:        **end if**14:    **end if**15:**end procedure**

Algorithm 4 shows a procedure when the $ME_{i}$ host receives a new task τ submitted by the requester $u_{r}$. First, $ME_{i}$ finds a *km* MGRS area in which τ is created and adds τ in the set of active tasks in this area (lines 2–3). Then, it checks if any of the workers who are currently located in the area can answer to τ (lines 4–5). If yes, $ME_{i}$ sends a current BF for this area and assigns τ to the worker $u_{w}$ (lines 6–7).
**Algorithm 4** Arrival of a new result in the area covered by the ME host1:**procedure**Receive Task($u_{r},\tau$)2:    $area\leftarrow findArea(\ell_{\tau })$3:    activeTasks(area).add(τ)4:    **for each** *i* in workersInArea(area) **do**5:        **if** $\mu_{\tau }\left(a\right)==⊤$ **then**6:           sendBF(bloomFilter(area),m)7:           $assign(u_{w},\tau )$8:        **end if**9:    **end for**10:**end procedure**

As already stated, a classic BF structure does not support the deletion of an element. As typical MCS service is time-dependent, i.e., sensor data becomes obsolete during the time, we overcome this problem by periodically resetting the BF structure maintained by an ME host, as presented in Algorithm 5. The $ME_{i}$ host periodically resets BF for its corresponding areas (lines 2–3) and finds potential workers in each area who will be activated upon receiving an empty filter (lines 4–6).
**Algorithm 5** Bloom filter reset on the ME host1:**procedure**Bloom Filter reset(bloomFilter)2:    **for each** area in bloomFilter.keySet() **do**3:        filterReset(bloomFilter(area))4:        **for each** *i* in workersInArea(area) **do**5:           sendBF(bloomFilter(area),m)6:        **end for**7:    **end for**8:**end procedure**

This section proposed a decentralized and edge-compliant architecture for MCS, which uses a well-known Bloom filter data structure to orchestrate autonomous data acquisition on mobile workers participating in MCS tasks. In the remainder of this paper, we extensively evaluate the proposed algorithm to determine if it can be used to reduce energy consumption in a distributed MCS environment.

## 4. Results and Discussion

In our previous work [9], we report on an initial evaluation of the BF approach. Hereafter, referring to [9], we further analyze our system with a real-world dataset to verify our initial findings that maintaining a BF structure does not incur a significant burden in terms of processing, memory, and bandwidth consumption and can be used for encoding MCS data in Section 4.1. In addition, we include novel results and evaluate the overall behavior of the proposed BF algorithm for different input scenarios by considering the overhead incurred in communication between ME host and end-user devices when the user changes its location or transmits redundant data readings in Section 4.2. We have also extended our initial solution so that multiple data readings of the same type can be accepted simultaneously on the user device and the ME host.

We used a real-world dataset presented in [10] for the evaluation purpose, which contains locations collected from 85 users in South Korea from March 2011 to September 2012. On average, a single user utilized a mobile application for tagging sites for 79 days. In total, users have tagged 13,500 different locations. The dataset was initially designed for autonomous place detection. Therefore, we had to modify it to model the motions of a broad user base suitable for MCS deployments. We note that the dataset does not include information about energy consumption. First, we initially filtered the dataset to exclude entries that did not have an accurate location or timestamp associated with them. There were 151,649 user check-ins from 67 different individuals in the filtered dataset. Because the number of unique users is too small to analyze a realistic MCS scenario, we separated the filtered set based on user identity and date. As a result, we generated numerous virtual user traces from a single genuine user’s trace for a single day. Each slice (a user-day slice is a sequence of user check-ins for a single day) represented a virtual user’s movement pattern for that day. Our evaluation is based on a total of 7724 virtual users and their accompanying user-day traces. We constructed additional check-ins (similar to the linear interpolation method presented in [51]) with a sampling frequency of 1 min by interpolating the expected user location between two consecutive check-ins because the data collection did not contain user location information with a high sampling frequency as required in MCS. That resulted in a realistic MCS data-trace with 4.7 million user-location records in total (on average, 600 location entries per user).

### 4.1. Evaluation of BF Parameters

As already stated in this section, referring to [9], we further evaluate a BF structure for the MCS domain with a different input dataset. In contrast to our previous work, where we have used just a tiny dataset containing 200,605 sensor readings (in total), hereafter, we use a dataset that is 23 times larger to thoroughly evaluate the applicability of BF structure in the MCS scenario containing realistic data traces. In Section 3.2, we have already shown that an optimal BF size and the number of hash functions can be calculated from the number of inserted elements *n* and the probability of false positives *p*. Therefore, in this part of the evaluation, we observe the behavior of a BF structure when encoding MCS data concerning those two parameters. Our goal is to identify distinct data readings which need to be inserted in a BF during its validity period (i.e., a period prior to filter reset), where a *distinct* reading is defined as a unique combination of parameter name and a *small* MGRS area identifier in the observed time slot. In other words, two data readings are redundant if they are collected in the same time slot (e.g., 5 min period), inside the same MGRS area of 100 × 100 m^2^ (i.e., *small* MGRS area) and associated with the same parameter (e.g., CO), and only one of them should be inserted in a BF structure during that period. A BF is reset upon the end of each time slot (e.g., 5 min period), meaning that a new empty BF structure, which will be used in the next time slot, is initialized. As we can assume the expected number of data readings that need to be inserted in the filter (i.e., *n*), and we can set the desired probability of false positives *p* for an MCS service, in all experiments, a filter structure is initialized with an optimal number of bits *m* and hash functions *k* calculated from Equations (4) and (5). Note that in our previous work described in [9], we have used just one filter for the evaluation purpose, meaning that all data readings have been inserted in the same BF structure, while in the remainder of this section, we use multiple BFs, one for each active *km* MGRS area following the proposed algorithm as described in Section 3.3. In addition, to give a reader true insight into the error rate caused by using the BF structure for encoding MCS data, it is not realistic to insert all of the 4.7 million data readings that have been placed in a single day (as previously described) in just one filter structure.

**Figure 4:**
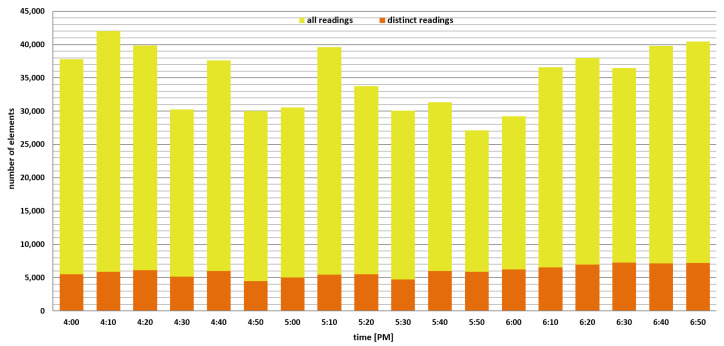
Ratio between all and distinct data readings in the period from 4 p.m. to 6:50 p.m.

**Figure 5:**
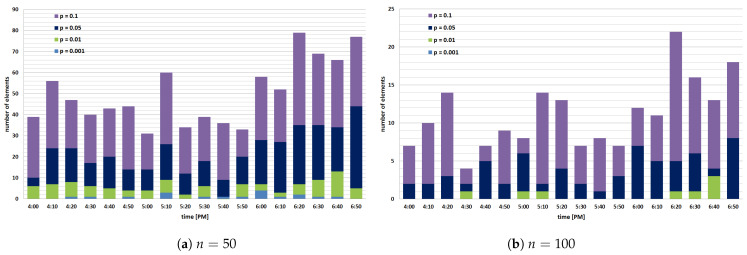
The influence of parameter *n* on the number of lost elements.

First, we investigate the impact of parameter *n* on the number of distinct data readings that were lost because the BF membership test failed and concluded that a reading (i.e., the combination of observed parameter and *small* MGRS area identifier) is already inserted in the structure (although it was not) when a predefined probability of false positives *p* is in the range from 0.1% to 10%. In the experiment, we use just a subset of data collected in the afternoon during a 3 h period between 4 p.m. and 7 p.m., as shown in Figure 4, and reset each BF structure every 10 min (i.e., a BF validity period is 10 min). Each yellow bar in the graph shows the total number of data readings in the observed time slot (e.g., there are 30,227 readings in the time slot from 4:30 p.m. to 4:40 p.m.), while the orange segment in the bar represents the total number of distinct data readings that must be inserted in BFs during this period. In general, overall users have gathered between 30 and 42 thousand data readings in each time slot, but a relatively small proportion of them are indeed valuable and should not be lost during the BF encoding process (i.e., between 4.4 and 7.2 thousand data readings are unique in each time slot, while other data readings are redundant and can be discarded). Figure 5 demonstrates how the parameter *n* affects the number of distinct (useful) data readings lost in each observed time slot. In particular, the number of lost elements is represented by blue, green, dark blue, and violet segments in the bar when the probability of false positives *p* was 0.1%, 1%, 5%, and 10%, respectively. As expected, when we compare the number of lost readings for different values of *p*, we can see that the lowest error is attained in every observed time slot when the probability of false positives *p* is 0.001 (i.e., 0.1%). Furthermore, when we compare Figure 5a,b, we can see that a larger value of the expected number of different elements *n* will considerably minimize data loss, even when *p* is 0.1, as a Bloom filter will be initialized with a greater number of bits and hash functions. For example, when *p* is 0.1, and *n* is raised from 50 to 100, the number of lost data readings drops from 79 (out of 6976) to 22 in a period between 6:20 p.m. and 6:30 a.m., while for p=0.001 it drops from 2 to 0 for the same time slot. In addition, as shown in Figure 5b, there are no lost elements in the entire observed period when p=0.001 and n=100; however, for n=50, the total number of lost elements is 18, as shown in Figure 5a. In general, we can conclude that if we initialize BFs with a higher *n* and lower *p*, we will obtain greater precision (i.e., higher accuracy) because the vector will employ more bits and hash functions for mapping elements (i.e., data readings).

**Figure 6:**
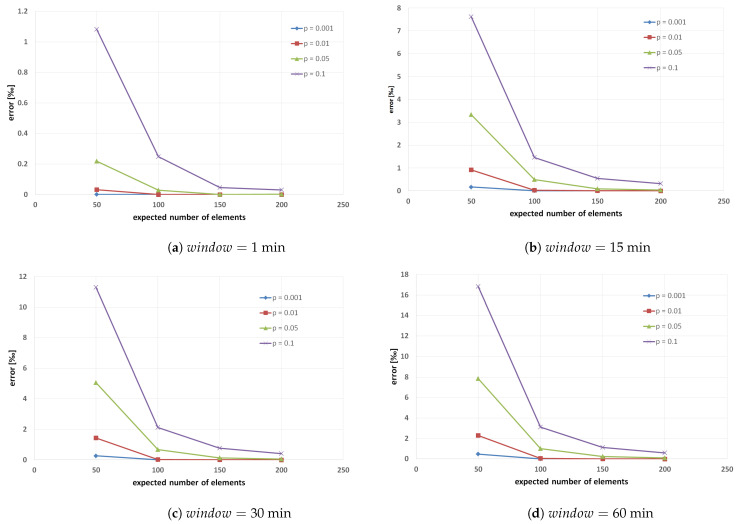
Percentage of elements that were lost during a 24 h period for different BF validity periods.

Then, we investigate how the duration of the filter reset period impacts the number of data readings lost because the BF membership test failed. More precisely, we analyze the total number of distinct elements (i.e., readings) that were lost throughout 24 h, depicted as an error rate (in per mille, ‰) in Figure 6, with regards to the BF validity period (i.e., each BF is periodically initialized at the beginning of every time slot throughout a day). It is critical to minimize this error as each of these lost elements is valuable and does not have a replacement in the observed time slot. As expected, we can see that the error rate is generally lower with a shorter window as the BF structure is more frequently reset. When we analyze the worst-case scenario, i.e., we expect to insert only 50 elements in the BF and the probability of false positives is 0.1 (i.e., 10%), around 1.1‰ of distinct data readings are lost throughout the day when the window duration is 1 min (see Figure 6a), while the error rate is round 7.6‰ when we reset BF every 15 min (see Figure 6b), and finally it goes up to 17‰ when BF reset period is every 60 min (see Figure 6d). In other words, although we allow having 10% of false positives, the actual error rate is significantly below this border because it goes from just 0.1% for a 1 min BF validity period up to 1.7% in the worst-case scenario when the BF validity period is 60 min. On the other hand, when we also expect to insert only 50 elements in the BF, but the allowed probability of false positives is a hundred times lower (i.e., 1‰), the actual error rate is less than 0.5‰ even when the window duration period is 60 min. Note that we expect to encode only 50 elements in each BF, but the actual number of inserted data readings is significantly higher in each time slot, and we are still below this very low allowed error rate, even when filters are reset every 60 min. As the BF validity period depends on the type of MCS service, our goal is to find parameters *n* and *p* that will keep the number of lost data readings to a minimum, regardless of the filter validity period duration. For example, noise levels need to be measured more densely than air pollutants, meaning that noise pollution monitoring will require a more frequent BF reset. It is self-evident that when *n* increases and the probability of false positives *p* decreases, the error rate will also decrease because the BF vector size is larger, and more hash functions are used for the mapping of elements (e.g., for n=50 and p=0.1 vector size is 239 bits and number of hash functions is only 3, while for n=200 and p=0.001 vector size is 2875 bits and number of hash functions is 10). As mobile phones also need to monitor Bloom filter structure in a distributed MCS architecture, we want to keep the vector size as small as possible while maintaining the number of hash functions to avoid losing too much data. Our analysis of a real dataset shows that this requirement can be met when the expected number of inserted elements *n* is 100 and the probability of false positives *p* is 0.001 (resulting in vector size of 1437 bits and ten hash functions), as the percentage of lost data readings will be around zero regardless of the BF validity period, as shown in Figure 6c,d, where the error rate is 0.006‰ and 0.008‰, respectively. Therefore, we use those values as BF input parameters in the remainder of our evaluation described in Section 4.2. Note that we did not use complex evaluation metrics such as area under the ROC curve (AUC) to evaluate BF because it does not apply to our approach as the BF data structure by definition does not contain false negatives, which is part of the receiver operating characteristic (ROC) curve.

**Figure 7:**
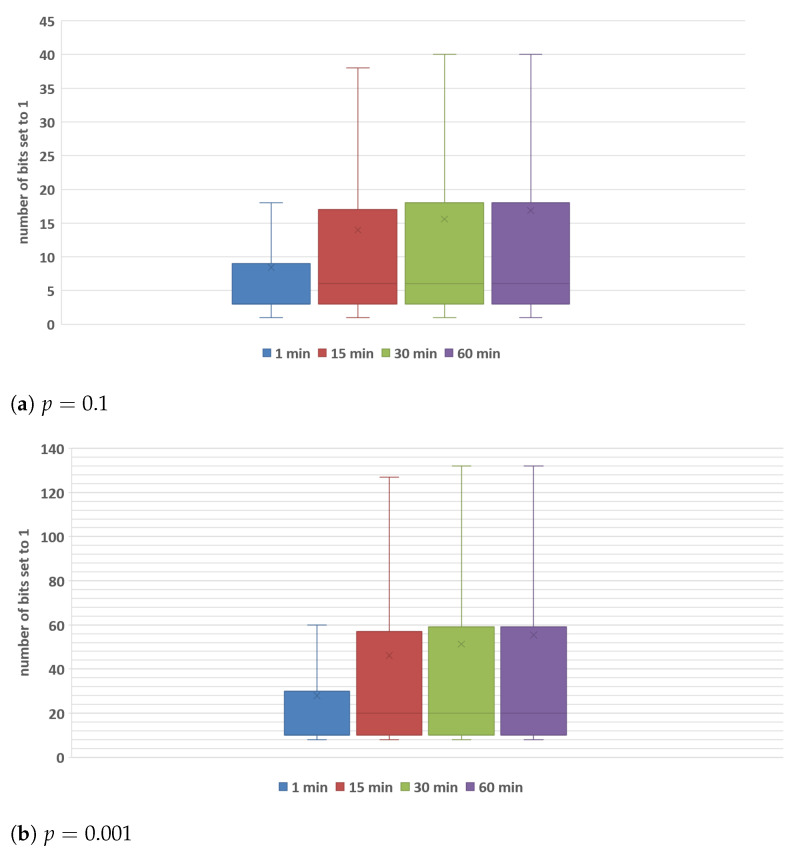
BF structure occupancy for different BF validity periods when n=50.

Finally, for each BF associated with an active *km* MGRS area, we analyze how many bits in the BF structure are set to 1, as setting too many bits to 1 can lead to an increased number of false positives, which can result in the lost data readings in our MCS service, i.e., measurements can be discarded because they were marked as redundant. Figure 7 presents the number of bits that have value of 1 when *n* equals 50 and parameter p=0.1 (Figure 7a) and p=0.001 (Figure 7b). We measured the BF occupancy (number of bits set to 1) for different time windows during 24 h to obtain the distribution of the number of bits that are set to 1. It is important to note that the total size of BF is 239 bits when the parameter p=0.1, and *m* is 718 bits for the scenario when the parameter p=0.001. We can see that the structure occupancy for each observed BF validity period is not close to the maximal number of bits. As expected, the results show that when the BF validity period is just 1 min, the structure occupancy rate is the lowest compared to longer BF validity periods because the least number of elements is encoded in the filter, and there is no significant difference in the BF structure occupancy for longer BF validity periods. In addition, it is interesting to observe that the cumulative distribution of occupancy per BF validity period is not primarily affected by the probability of false positives *p*, i.e., Figure 7b does not change its shape significantly compared to Figure 7a. That means that the occupancy ratio remains stable despite the increase in the BF size (only absolute numbers correlated to the number of hash functions are affected). Even though the presented graphs show that the maximal number of bits set to 1 is not going beyond 40 (Figure 7a) and 132 (Figure 7b), one should note that there are some points located outside the whiskers of the box plot (i.e., above the ninth decile which are not depicted on the graph). These points indicate that in some periods during a day, some individual filters have more bits set to 1 due to the increased number of data readings encoded in the BF, causing that actual BF error to be greater than the allowed probability of false positives. For example, when the filter validity period is 15 min, n=50 and p=0.1, there is a *km* MGRS area with 100 distinct data readings in the observed period of which 85 are successfully encoded in the BF, and the filter has 174 out of 239 bits set to 1 resulting in an error higher than allowed. However, one should also note that in this specific case, 1.7 times more data readings were encoded in the BF than expected (i.e., BF expected to receive maximum n=50 elements). We have confirmed our initial findings from [9] that the BF overall size and memory footprint allow it to be employed on tiny devices, indicating that the structure is suitable for mobile devices to determine whether or not reading should be transmitted to an ME host.

### 4.2. Evaluation of BF Algorithm

One of the main characteristics of our decentralized MCS ecosystem is that it suppresses redundant and possibly irrelevant data readings by using the BF structure to deactivate some of the workers. However, this is achieved at the expense of filter control messages. Therefore, in this section, we first overview the messages exchanged between workers (i.e., their mobile devices) and ME hosts in our solution and then evaluate the proposed algorithm on a real-world dataset.

As already stated in Section 3.2, an ME host is deployed in an MGRS area of 10 square kilometers and maintains BFs for all *km* MGRS areas under its responsibility. It is aware of all user requests (tasks) and workers in the area. Whenever a worker moves to a neighboring *km* MGRS area, it will send an *announce* message to the corresponding ME host and receive an *assign* message as a reply if at least one task is assigned to a worker, and it will start a data acquisition process and *submit* results to the ME host until its BF membership test reports a positive match. In case the corresponding ME host determines that the submitted result is redundant (i.e., it has already received all required data readings in the meantime), it will send a BF *update* message to the worker who submitted this result. Additionally, a BF maintained by the ME host is periodically *reset* and delivered to the potential workers in the area. Therefore, the total number of exchanged messages equals the sum of all messages explained above (see Equation (7)):

(7) $$M_{MEC}=M_{ann}+M_{ass}+M_{sub}+M_{BFupdate}+M_{BFreset},$$

where $M_{MEC}$ is the total number of messages that are exchanged within our MCS ecosystem, $M_{ann}$ is the number of *announce* messages, $M_{ass}$ is the number of *assign* messages, $M_{sub}$ is the number of *submit* messages, $M_{BFupdate}$ is the number of BF *update* messages, and $M_{BFreset}$ is the number of BF *reset* messages in the system.

Next, we observe the possible energy savings achievable with our approach compared to the baseline approach when all results produced by workers are transmitted to the ME hosts. Given that the energy consumption is in a linear relationship with the number of generated messages, the savings can be calculated as the percent decrease in the number of transmitted messages generated by our solution as compared to all produced results (see Equation (8)):

(8) $$Savings_{MEC}=\frac{M_{allResults}-M_{MEC}}{M_{allResults}},$$

where $Savings_{MEC}$ is the energy savings, $M_{allResults}$ is the number of all produced results that are transmitted in a traditional baseline MCS system, and $M_{MEC}$ is the total number of messages that are exchanged within our MCS ecosystem.

We analyze the behavior of our solution concerning parameters $m_{worker}$, $m_{MEC}$, and window, where $m_{MEC}$ indicates a required number of results (of the same type) per *small* MGRS area of 100 m^2^ under the authority of an ME host, $m_{worker}$ indicates the number of results that a worker needs to submit per each *small* MGRS area of 100 m^2^ before updating its BF, and window indicates duration of the observation time slot in which a BF structure on the ME host is valid prior to reset (i.e., a day is divided into sequential independent time windows with a fixed duration, and upon the end of each time slot, a BF structure is reset). The initial parameter configuration is shown in Table 1. Note that the number of all produced results, as well as the number of *announce* messages, are obtained from the dataset, while the default values chosen for $m_{worker}$, $m_{MEC}$, and window are hypothetical. The actual values for these three parameters in a real-world case would depend on the geographical configuration of a city, its population distribution, user mobility, and implemented application.

**Figure 8:**
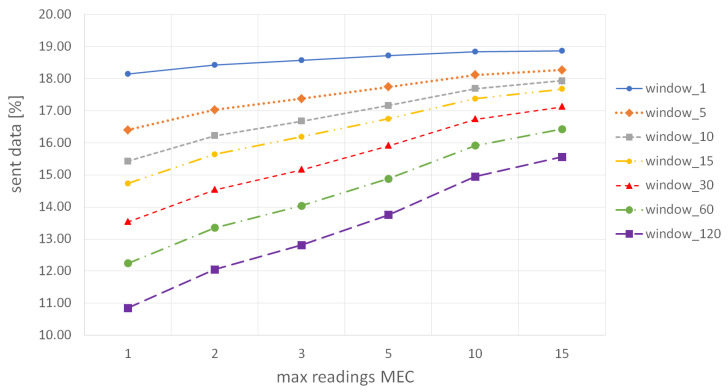
Percentage of data sent by all workers when $m_{worker}=1$.

First, we analyze the influence of parameters $m_{MEC}$ and window on the amount of sent data when the parameter $m_{worker}$ has a fixed value of 1 (i.e., a worker updates its BF structure after submitting the first result in the *small* MGRS area). In other words, Figure 8 depicts the cumulative number of results that were sent by all workers in our distributed system when an MCS service deployed on the ME host requires up to 15 results per each *small* MGRS area, and each worker is instructed to submit only one result per *small* area. As expected, the results show that with a longer window duration (i.e., the BF validity period), the number of sent results will be lower because the time between two consecutive filter resets is longer, and workers discard more results. In particular, when the filter reset period is 1 min, the percentage of *useful* data sent by all workers is between 18% and 19%, while with the reset period of 120 min, this percentage drops to values between 11% and 15.5%, depending on the required number of workers per *small* MGRS cell. We can see that when $m_{MEC}$ has the value of 1 (i.e., ME host requires only one result per every 100 m^2^), the amount of generated data is the lowest, because hosts update their BF immediately after they receive the first result for the *small* MGRS area. In contrast, for bigger $m_{MEC}$, hosts expect to receive more results and update their filters less often, which leads to a higher amount of data sent by workers. When the filter validity period is 1 min, the influence of the parameter $m_{MEC}$ on the percentage of sent data is not significant because there are not many workers located in the same area in such a short time interval, and the amount of redundant data is similar regardless of the required amount of data per *small* MGRS area. On the other hand, when filter reset occurs every 120 min, it is very likely to find more workers in the same area who can submit data during the observed period, which results in greater differences in the percentage of sent data for different values of the parameter $m_{MEC}$. For example, when $m_{MEC}$ is 1, the required amount of data on the ME host is collected very soon. Suppose other workers come to the same area in the remaining period. In that case, they will be instructed not to produce measurements because they receive an updated version of a BF (i.e., the host has already inserted received results in the filter), while when $m_{MEC}$ is 15, more workers who send an announcement later in the time slot will also receive an initial (empty) version of the BF, and the ME host will receive more data readings. Overall, we can conclude that more than 80% of data readings produced by all workers in the system are never sent to edge hosts even in the worst-case scenario when the filter validity period is only 1 min, and MCS service requires up to 15 results per each *small* MGRS area. That is a significant reduction in the number of transmitted data compared to a baseline approach where all produced data readings are sent to the cloud server.

**Figure 9:**
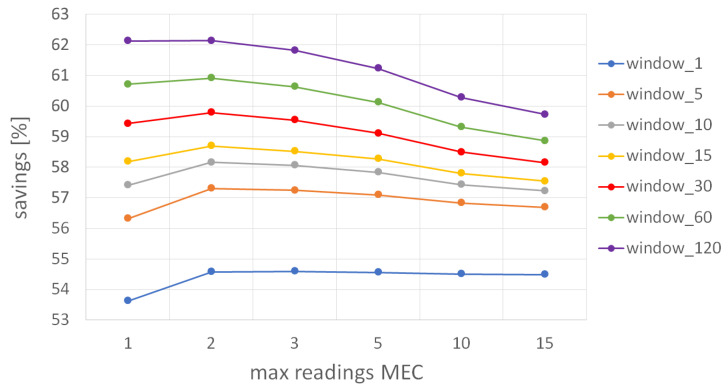
Energy savings when $m_{worker}=1$.

Next, we observe trends in energy savings (8) with respect to parameters $m_{MEC}$, $m_{worker}$, and window. We analyze the number of all transmitted messages in our distributed MCS architecture compared to the baseline approach in which all produced data readings would be sent to the edge hosts. We modify two parameters for each analysis, while the third parameter is fixed to one of the default values given in Table 1. First, in Figure 9, we show how parameters $m_{MEC}$ and window influence the savings when workers are instructed to transmit only one data reading per 100 m^2^. In general, the results indicate that the advantage of our approach drops when increasing the value of parameter $m_{MEC}$ because the required number of workers per *small* MGRS area increases, consequently leading to a greater number of submitted results per cell, as already shown in Figure 8. If more workers are required per *small* area by ME host, worker self-deactivation by updating their BF has less influence because there is a need to satisfy application requirements (i.e., ME hosts need to collect $m_{MEC}$ results before updating their filters). In addition, the results show that savings are generally lower when the BF validity period is shorter due to the increased number of transmitted data readings between workers and ME hosts. Although one might expect that energy savings will follow trends in the number of results submitted by workers (i.e., when the number of useful data increases, the total savings drops), we can see a slight deviation when $m_{MEC}$ is one due to the overhead introduced by filter update and reset messages. Whenever a worker submits a redundant result to the corresponding ME host, it receives a *global* filter for the *km* MGRS area where it is currently residing (i.e., an updated BF which contains all results received by the ME host in the area), and when ME hosts require just one result per *small* area, this can occur quite often. That is especially visible for short filter validity periods (e.g., 1 min) when the number of reset messages also increases. Overall, we can see that the total savings are above 53% even in the worst-case scenario when the filter validity period is just 1 min, while it can go up to 62% if filter reset occurs every 120 min.

**Figure 10:**
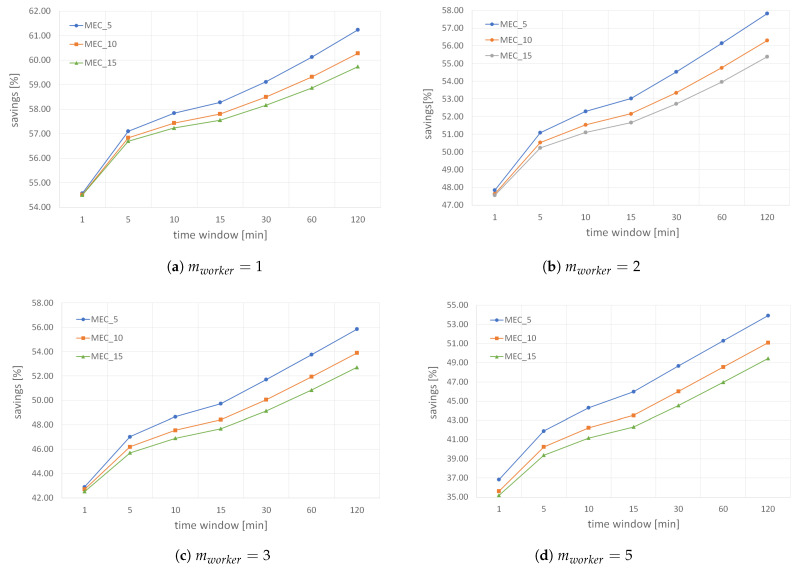
Energy savings with respect to the parameter $m_{worker}$.

Figure 10 shows how BF validity period and MCS application requirements influence the total energy savings when the required number of results, which a worker has to produce per *small* MGRS area before updating its BF (i.e., $m_{worker}$), is fixed to one of the default values from Table 1. The results confirm that savings drop when ME hosts require more data readings per *small* MGRS area and the time between consecutive filter resets is shorter. In particular, the results indicate that a filter validity period of just 1 min significantly reduces the total saving compared to longer filter validity periods (e.g., 5 min) due to the overhead caused by many reset messages. Furthermore, when we compare the total savings in Figure 10a,d, we can see that savings significantly drop with higher values of $m_{worker}$. If workers are instructed to transmit more results to the edge host, then filtering mechanisms have less influence to reduce the total savings. However, even in the worst-case scenario shown in Figure 10d, when we need high redundancy per *small* MGRS area (i.e., up to 5 results for every 100 m^2^), the energy savings is around 35% when the time between consecutive filter resets is just 1 min, and can grow up to 55% when filter validity period is 120 min.

**Figure 11:**
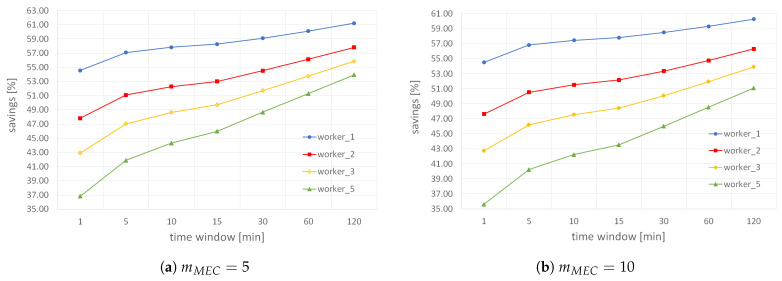
Energy savings with respect to the parameter $m_{MEC}$.

Next, we analyze how the number of results a worker needs to submit per *small* MGRS area and the length of BF validity period influence the total savings while keeping the fixed value of $m_{MEC}$, as shown in Figure 11. The results indicate that when increasing the time between consecutive filter resets (i.e., window), the influence of the parameter $m_{worker}$ drops. In other words, when the filter validity period is just 1 min, the difference in savings for $m_{worker}=1$ and $m_{worker}=5$ is around 18% (i.e., the total savings is 18% lower when workers are instructed to submit five results per *small* area), while this difference drops to only 7.5% when filter reset is every 120 min when the parameter $m_{MEC}$ has a fixed value of 5. Similarly, when $m_{MEC}$ is 10, the difference in savings for $m_{worker}=1$ and $m_{worker}=5$ is around 18.5% when window is 1 min, and drops to 9.3% when filter validity period is 120 min. In addition, we can see that the total savings slowly increases with a decrease in the value of parameter $m_{MEC}$ because an MCS application deployed on ME hosts requires fewer results. In particular, when workers submit only one result per *small* MGRS area and the filter validity period is 120 min, the total savings goes up to 61.2% when $m_{MEC}$ is 5 (see Figure 11a), while it is less than 1% lower when ME host needs up to 10 results per *small* area (see Figure 11b). That is a significant result because savings remain pretty high even for many data readings required by an ME host.

Our findings are summarized in Table 2 and Table 3, which show the absolute difference in total savings for different input parameters. The first table shows the influence of parameter $m_{worker}$ on total savings, where column $m_{worker\_x,y}$ presents the absolute difference in savings (expressed as percentage) when we compare the savings result for $m_{worker}=x$ and $m_{worker}=y$ for different values of parameters window and $m_{MEC}$ (e.g., the result in the first row of column $m_{worker\_1,2}$ shows that the total savings is 6.73% higher for $m_{worker}=1$ when filter reset is every 1 min and ME host requires 5 results per *small* area), while the second table shows the influence of parameter $m_{MEC}$ on total savings, where column $m_{MEC\_x,y}$ presents the absolute difference in savings when $m_{MEC}=x$ and $m_{MEC}=y$ for different values of parameters window and $m_{worker}$ (e.g., the result in the fifth row of column $m_{MEC\_5,10}$ shows that the total savings is 0.95% higher for $m_{MEC}=5$ when filter reset is every 120 min and worker submits only 1 result per *small* area). The results indicate that the parameter $m_{worker}$ has significant influence on the total savings when compared to the parameter $m_{MEC}$. We can conclude that $m_{MEC}$ is used to control the number of workers who can produce data in each *small* MGRS area during an observed time slot (i.e., BF validity period), while $m_{worker}$ is used to control the amount of data that each worker is going to produce per area. In other words, the significance of the parameter $m_{MEC}$ is manifested only in cases when many redundant workers are located in the same 100 m^2^ area during the same period.

**Figure 12:**
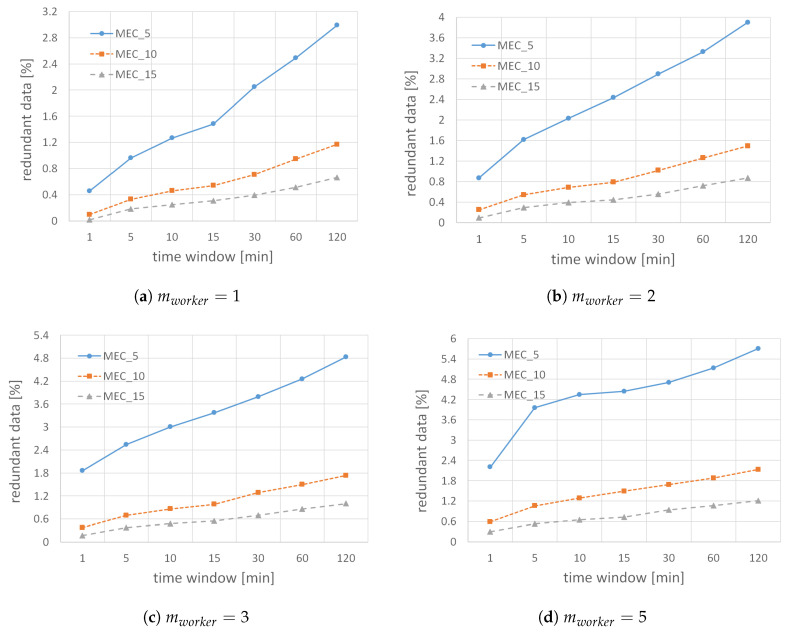
Percentage of redundant data received by ME hosts with respect to the parameter $m_{worker}$.

Finally, we analyze the influence of parameter $m_{worker}$ on the cumulative amount of redundant data received by all ME hosts for different filter reset periods and application requirements in Figure 12. As already stated, a data reading is redundant if an ME host already contains the same combination of the observed parameter and *small* MGRS area identifier in its BF structure. Note that the percentage of redundant results is expressed concerning the total data received by ME hosts. As expected, the highest percentage of redundant data is received when hosts require only five data readings per *small* cell before updating the global filter for the area. The lowest amount of redundant data is received when hosts require 15 results for all observed scenarios, regardless of the time window in which BF is reset and parameter $m_{worker}$, because for smaller values of $m_{MEC}$, hosts will update their filters very soon, and subsequently received results will be redundant. When we compare savings for different BF validity periods, we see that the amount of redundant results received by ME hosts grows when the time between BF resets increases. If the filter is valid only for a short period, the probability that more workers will be within the same *small* MGRS area during the same time slot is low. In other words, likely, hosts will not collect the expected number of results per each *small* area because the filter will quickly reset. At the same time, it is quite possible that within, for example, 120 min, more workers will pass through the same *small* area, and because of the relatively high values of the parameter $m_{MEC}$, a filter they receive may still not be updated with recently received results, which may later cause a more significant amount of redundant data in that area.

Similarly, when we compare the percentage of redundant results received by ME hosts for different values of the parameter $m_{worker}$, we can see that the amount of redundant results grows with $m_{worker}$ because workers are instructed to produce more readings per *small* area and need to wait longer before updating their filters. Therefore, a worker may send a redundant result because its version of the filter shows that this result is valuable and should be submitted, while in the meantime, the corresponding ME host has already received enough results for that *small* area from other workers. Overall, the results show that even in the worst-case scenario, when workers are instructed to submit five results per each *small* area, as shown in Figure 12d, the percentage of redundant results received by ME hosts never exceeds 6%, which proves that the proposed algorithm works exceptionally well for different input scenarios.

To conclude, we have shown that our hierarchical edge computing architecture, enhanced with the algorithm for autonomous decision-making of a mobile worker, shows great potential for energy savings in distributed MCS environments while retaining the desired sensing quality due to decreased messaging between workers and ME hosts. The main advantage of the proposed hierarchical architecture is that there is no central point of failure because the data processing is distributed across multiple ME hosts, and all workers can autonomously decide when to transmit data. At the same time, the actual savings depend on an actual MCS application and its setup. Our analysis showed that the edge architecture is suitable for massive-scale MCS services. The amount of transmitted data between mobile workers and edge hosts can be significantly reduced using the proposed BF algorithm because workers independently decide when to send data instead of periodically transmitting all data readings. Consequently, it is possible to achieve energy savings of up to 62%.

## 5. Conclusions

Mobile crowdsensing refers to a set of human-driven IoT applications that allow users to detect phenomena of personal, communal, or even societal importance by sharing sensor data about their surroundings while being mobile. Typical MCS service deployment has a cloud-based centralized design that requires many computing resources and creates a lot of network traffic, both on mobile networks and towards cloud-based MCS services. Furthermore, MCS applications produce large amounts of data collected and preprocessed by devices with limited energy supply. Hence, there is a need for solutions that will reduce the energy consumption of such devices while satisfying application requirements in terms of the quality of acquired datasets.

This paper proposes a hierarchical MCS deployment suitable for edge computing environments. Because ME hosts are responsible only for workers and MCS tasks inside their deployment area, such architecture allows parallelizing and segmenting the problem space based on the location. In particular, we propose a decentralized algorithm that allows mobile workers to engage in data collection and transmission processes on their own (i.e., independently) without requiring cloud-based supervision and coordination. The proposed algorithm uses a Bloom filter structure, both on mobile nodes and ME hosts, to minimize redundant worker activity while at the same time keeping uniform data density within the area. We evaluate the BF algorithm on a real-world dataset and show that it is a valuable data structure to orchestrate autonomous data acquisition on workers participating in MCS tasks. In particular, our results indicate that the proposed approach shows great potential for energy savings in distributed MCS environments due to decreased messaging between workers and edge hosts while satisfying application requirements. We have shown that the overall energy savings go from 35% up to 62% compared to a baseline approach. Because data processing splits over many ME hosts and all workers may autonomously determine when to send data, the proposed hierarchical edge-based MCS deployment has no single point of failure. However, the actual energy savings are highly dependent on the MCS application and its setup.

We have proposed a generic solution for a distributed data collection in the MCS system, but additional savings can be achieved if the algorithm considers application-specific requirements. For example, in some cases, it could be better to reset BF after it has been filled to a certain extent instead of periodical reset in predefined time intervals. Such algorithm adjustment can further reduce communication between ME hosts and mobile workers, as filter reset messages are sent when required. Finally, we plan to investigate user traces obtained from cellular network providers to obtain a better insight into user mobility patterns and identify *popular* areas. In areas with many workers, one can expect high data redundancy, especially after BF reset, because all workers would simultaneously start data transmission to the corresponding edge host. Additional savings can be achieved by choosing only top-*k* workers in the area, based on a valuation function, who will receive an empty filter after reset. Such an approach would reduce the amount of sent data and messaging between edge hosts and workers in the area and consequently increase system energy savings.

## Figures and Tables

**Table 1:** Default parameter values.

Symbol	Values
*allResults*	4,709,259
announcements	579,040
$m_{worker}$	1; 2; 3; 5
$m_{MEC}$	1; 2; 3; 5; 10; 15
window (in minutes)	1; 5; 10; 15; 30; 60; 120

**Table 2:** Influence of the parameter $m_{worker}$ on the total savings.

*Window*	$m_{\mathrm{MEC}}$	$m_{\mathrm{worker}\_1,2}$	$m_{\mathrm{worker}\_2,3}$	$m_{\mathrm{worker}\_3,5}$
1 min	5	6.73%	4.91%	6.09%
	10	6.86%	4.91%	7.11%
	15	6.93%	5.02%	7.34%
120 min	5	3.41%	1.96%	1.93%
	10	3.98%	2.39%	2.92%
	15	4.34%	2.68%	3.27%

**Table 3:** Influence of the parameter $m_{MEC}$ on the total savings.

*Window*	$m_{\mathrm{worker}}$	$m_{\mathrm{MEC}\_5,10}$	$m_{\mathrm{MEC}\_10,15}$
1 min	1	0.06%	0.01%
	2	0.19%	0.08%
	3	0.19%	0.19%
	5	1.21%	0.42%
120 min	1	0.95%	0.55%
	2	1.52%	0.91%
	3	1.95%	1.20%
	5	2.84%	1.65%

## Data Availability

The data presented in this study is described in [10] and is available at http://lifemap.yonsei.ac.kr/ubicomp13.htm (accessed and downloaded on 2 June 2014).
